# Treatment of Exposed Pulps by Lacto Phosphate of Lime

**Published:** 1876-10

**Authors:** Junius E. Cravens

**Affiliations:** Indianapolis, Indiana.


### ARTICLE VI.

### *Lacto-phosphate of Lime; Pathology and Treatment of Exposed Dental Pulps and Sensitive Dentine.*

BY JUNIUS E. CRAVENS, D. D. S., INDIANAPOLIS, INDIANA.

An idea is apt to so envelop the originator that he sees but one side of it, so that I may hope to be leniently judged if I have failed to view the outside of a theory of practice the honor of whose paternity has been accredited to me.

My experiments with lacto-phosphate of lime began in the winter of 1872-73, in topical treatment of sensitive and soft or demineralized dentine, and pulp exposures. Previous to that time I had tried various methods of dealing with this knotty little problem, principally applying carbolic acid in various solutions.

My failures and otherwise with carbolic were not unlike those of other operators in character or numerical proportion: but so bad was that proportion that I felt constrained to abandon a practice that, so far as the delicate tissue treated was concerned, was little short of the *moxa*, and so fruitful of unsatisfactory results.

Even when a student of dentistry it was not very lucid to me why so violent and destructive an agent as carbolic acid or its equivalent should be so generally resorted to in efforts to preserve alive the exposed pulp; and this question became even more opaque when the self-same drug began to be used for the extermination of the pulp by those practitioners who proposed to preserve it by the same means. In this connection I am reminded of the fabled anchorite who expelled the belated traveler from his hermitage on a stormy night because the stranger warmed his benumbed fingers by blowing upon them, and directly cooled his soup by the same means. The recluse declared he would shelter no man who blew hot and cold with the same breath.

Carbolic acid is not a constituent of dentine, neither is cork nor gutta percha. Nature may tolerate them under certain circumstances, but it is already a forced compliance. When the pulp has been exposed it does not ask for any caustic agents, it is already too near destruction. What it does want is bone-material, food that may be converted into ossific matter ; pabulum, if you please, to assist in replacing lost structure.

The original covering of the pulp was dentine ; the principal constituents of dentine are cartilage, organic, and phosphate of lime, inorganic, matter; the former being formed within the body, whilst the latter must be obtained from external sources. From this point of observation it seemed to me that the phosphate of lime was the proper material to be supplied by the dentist, but how or in what form to present it was my dilemma. It is true the alimentary canal had been suggested, but then life is entirely too short to render that route practicable to supply the pulp with sufficient phosphate of lime to enable it to cover its own head.

A certain medical journal contained an extract of a paper written by one M. Locke on " Syrup Calcis Lacto-Phosphatum." M. Locke's idea was to predigest the phosphate in lactic acid before introducing it into the alimentary canal. If predigesting rendered this bone salt in a fit condition for the absorbents and for assimilation, why not carry it directly to the pulp, in sufficient quantity, already for assimilation whenever it enters in solution with the plasmic element of the blood? When the pulp is punctured, a drop of blood exudes; when the drop of blood clots, a contraction of fibrin forces plasma to the surface. This liquor sanguinis, the fluid element of blood, holds in solution the salts that are distributed by the circulation throughout the body. This fluid is the mother of all nutrition, of all development, and any substance that is to be assimilated into animal tissue must first make peace with the blood plasma. Therefore, when the plasma comes to the surface of the clot over the exposed pulp, give to it the lacto-phosphate of lime. I believe that assimilation will take place locally, and that nature will utilize the material given, and by her mysterious process of development stretch a bony covering over the exposed surface of the pulp. My first experiment demonstrated this, and subsequent observations have only served to confirm the opinion.

When the lacto-phosphate of lime is applied to an exposure of pulp, it should be carefully sealed up with os-artificial, and left undisturbed for several weeks. One application usually suffices for small exposures; large ones may require more. So for much exposed pulps; we will now pass to the consideration of *sensitive dentine.*

I do not know exactly to what pathological cause dental hyperæsthesia is attributable, but when I find this exalted sensibility I usually observe the softened consistency of the cavity parietes. Softened dentine is rendered so by the loss of its inorganic constituents, therefore I believe extreme sensitiveness of dentine to be due to demineralization. When equilibrium of organic and inorganic constituency of dentine is preserved, sensibility will remain normal. Now, as the mineral or animal element predominates, just so will the sensibility of dentine be obtunded or exalted. My treatment of sensitive dentine is based upon the above diagnosis. You may say that I use logic to bolster up my doctrine, but then, how often is it the case that logic is medicine's only friend!

In order to secure a paste consistency of lacto-phosphate of lime, I add to it enough of the powder phosphate to thicken as desirable. The paste thus formed will harden in the cavity of the tooth after a time, and the longer it is left undisturbed the harder it becomes, until a maximum is reached.

In excavating, care should be observed that all softened dentine be left undisturbed, except to dry it with some bibulous substance. The lacto-phosphate of lime will infiltrate and occupy this soft, fibrous-looking tooth-bone, and, hardening there becomes intertubular substance, not unlike that which was lost; it also enters the ends of the softened and enlarged tubuli, and, hardening there, effectually plugs them. Thus we have secured density and insensibility by establishing a preponderance of mineral where the organic element was in excess.

In soft white dental caries there will be observed in deep cavities a mass of spongy sticks or fibers lying in the base of the cavity. The dentist notes its moist, spongy character, and cuts it away; the pulp is thereby exposed, because nature left that pile of sticks as a last barricade against the enemies in the cavity of caries.

Now, if instead of removing the demineralized dentine that still stands sentry over the pulp you would simply absorb its moisture, and dress the cavity with the paste of lacto-phosphate of lime, after a week or more you will discover that the demineralized sticks and soft fibers have taken unto themselves hardness and density, and that you have simply utilized and perfected a capping provided by Dame Nature herself.

If any fluids of the mouth gain access to the lacto-phosphate in the cavity, a violent toothache is liable—even probable to ensue, so that it is best in every instance to first adjust the rubber dam. The saliva should never be allowed to enter the cavity from the time this treatment begins until completed by filling.

I do not believe that the dental pulp is exposed in over five per cent. of cases by caries, and then only by that known as soft white decay ; the ninety-five per cent. of exposures being made by the excavator or caused by external violence. So firm have I become in this belief that I seldom expose pulps of late, and consequently have very few upon which to experiment.

To those who are desirous of experimenting with or trying this method of treatment of pulp-exposures and sensitive dentine, I would suggest that they get from some manufacturing chemist the phosphate of lime in the *magma*, which is its soluble condition ; a small box of powder or dry phosphate of lime ; and some of Merck's lactic acid The *magma* should be bottled and covered with water, and always kept so, and tightly corked. The bottle of lactic acid should be tightly stopped. A couple of drops of the lactic acid will dissolve about the same quantity of the *magma* ; the solution is effected by rubbing in a few minutes, and is known as lacto-phosphate of lime. To this solution add the powder to any consistency you may desire and apply as I have directed.—*New York Odontological Society.—Cosmos.*

---

